# The complete chloroplast genome sequence of *Orostachys minuta* (Crassulaceae)

**DOI:** 10.1080/23802359.2022.2133556

**Published:** 2022-10-25

**Authors:** Ha-Rim Lee, Bo-Yun Kim, Halam Kang, Sung-Mo An, Kyung-Ah Kim, Kyeong-Sik Cheon

**Affiliations:** a Department of Biological Science, Sangji University, Wonju, South Korea; b Plant Resources Division, National Institute of Biological Resources, Incheon, South Korea; c Environmental Research Institute, Kangwon National University, Chuncheon, South Korea

**Keywords:** Crassulaceae, *Orostachys*, chloroplast genome, phylogeny

## Abstract

The chloroplast (cp) genome sequence is determined and analyzed for *Orostachys minuta* for the first time. The cp genome was 150,369 bp in length, containing a large single-copy (LSC) of 82,795 bp and a small single-copy (SSC) of 16,854 bp, which were separated by a pair of 25,360 bp inverted repeats (IRs). The overall G + C content of the *O. minuta* cp genome amounted to 37.7%. In total, 113 unique genes were annotated, consisting of 79 protein-coding genes (PCGs), 30 transfer RNAs (tRNAs), and four ribosomal RNAs (rRNAs). Among these genes, 18 contained one or two introns. A maximum-likelihood (ML) phylogenetic analysis based on 33 taxa showed that *O. minuta* formed a clade with *O. japonica*. This study will provide a baseline as well as valuable molecular phylogenomic information for various future studies to determine the taxonomic position and phylogenetic relationships of the genus *Orostachys*.

## Introduction

**Figure 1.**
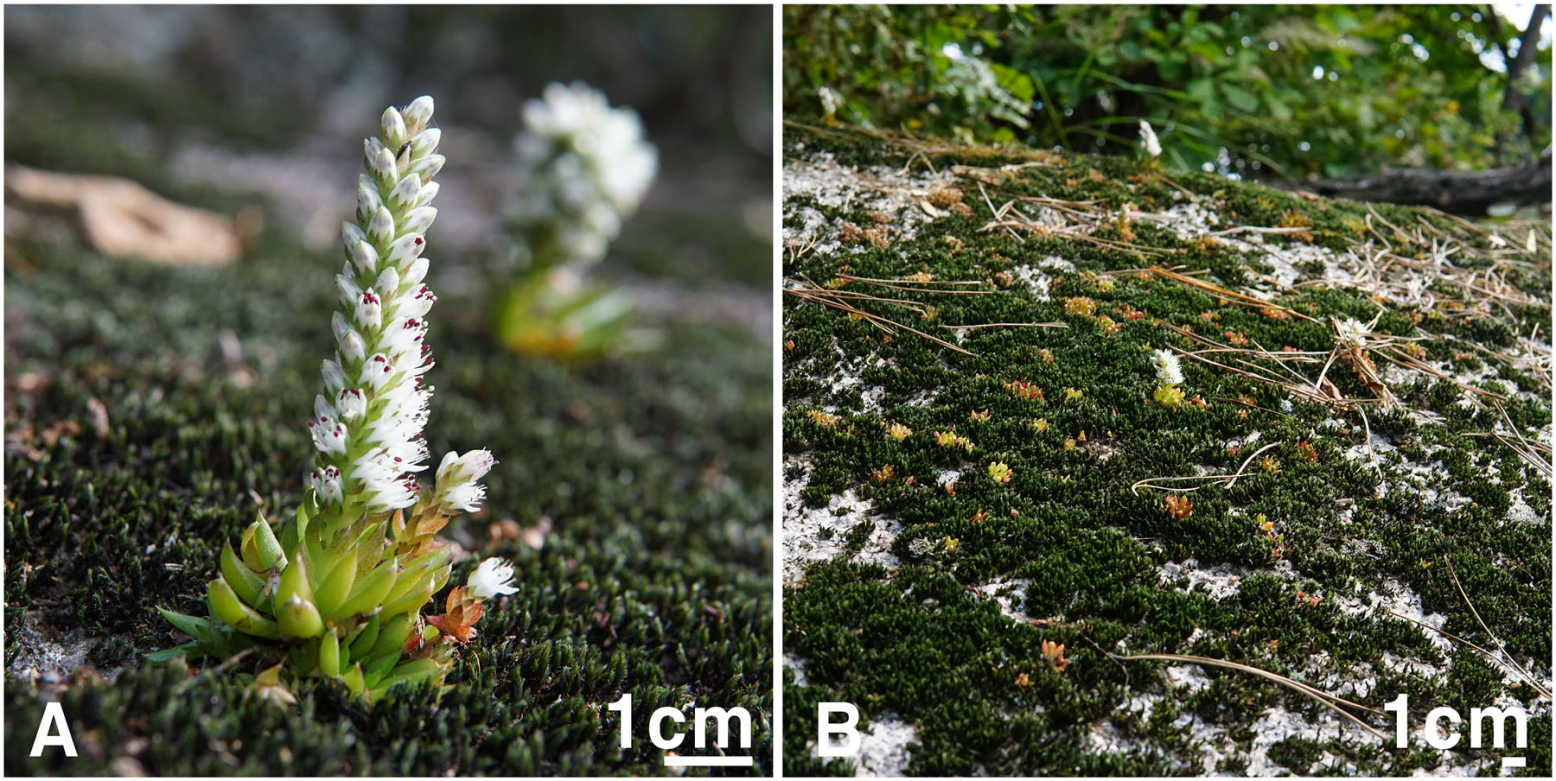
Photograph of *Orostachys minuta* (A) and its natural habitat (B).

The genus *Orostachys* Fisch. belonging to Crassulaceae includes approximately 20–25 taxa that are distributed from the Ural Mountains to Japan (Ohba 1990; Lee et al. 2003; Kozyrenko et al. 2013). This genus has traditionally been used as an ornamental and a medicinal plant, and some species have recently been shown to be effective in antioxidant and anticancer treatments, therefore, becoming recognized as a very important plant resource (Kim et al. 2003; Park et al. 2018). Among these, *Orostachys minuta* Berger, 1930 (Figure 1) is narrowly distributed on the Korean Peninsula and in China, with morphological characteristics of a flowering stem of 2–5 cm, leaves that are ovate-lanceolate, and spiny leaf apices. It was initially described as *Cotyledon minuta* (Komarov 1901) but has since been treated as a species belonging to the genus *Orostachys* (Berger 1930). Since species belonging to *Orostachys* have very similar morphological characteristics among species, this is a notoriously difficult group to classify. For this reason, although various studies have been conducted, the taxonomic position and phylogenetic relationship of many species are not clear (Mayuzumi and Ohba 2004; Kim and Park 2005; Gontcharova et al 2006; Gontcharova and Gontcharov 2009; Kozyrenko et al. 2013; Messerschmid et al. 2020). In this study, we report the first complete chloroplast (cp) genome of *O. minuta*, and conduct a phylogenomic analysis with other related species within Crassulaceae based on cp genome sequences.

## Materials and methods

*O. minuta* is not an endangered or protected species. We did not collect plant material from any privately owned or protected area that required permission. Ethics approval for this study was obtained from the Institutional Ethics Committee of Sangji University. The plant materials for this study were sampled from Jangheung-ri (38°11′52.2″N, 127°16′44.5″E), Cheorwon-gun, in Gangwon-do province in South Korea, and a voucher specimen was deposited at the Kangwon National University Herbarium (voucher no. KWNU100079; KA Kim, 1seizetheday@kangwon.ac.kr). Total DNA was extracted using a DNeasy Plant Mini Kit (Qiagen Inc., Valencia, CA). Paired-end sequencing for the cp genome of *O. minuta* was performed on the Miseq (Illumina Inc., San Diego, CA) platform. We obtained 1,293,824 raw reads with a length of 301 bp. The assembly and annotation of the cp genome were accomplished using the Geneious prime^®^ v.2021.1.1 software package (Biomatters Ltd., Auckland, New Zealand). We also compared each gene to the published complete cp genome sequence of Crassulaceae for correct gene annotation (Chang et al. 2021). The transfer RNAs (tRNAs) were confirmed using tRNAscan-SE (Schattner et al. 2005).

To construct the phylogenetic tree, the complete cp genomes of 32 species were selected within the subfamily of Sempervivoideae in Crassulaceae. One additional species from the subfamily Kalanchoideae (*Kalanchoe tomentosa*) was chosen as an outgroup. The cp genomes of the 33 accessions were aligned using the MAFFT (Katoh and Standley 2013) plugin of the software Geneious prime^®^ v.2021.1.1 (Biomatters Ltd., Auckland, New Zealand). A maximum-likelihood (ML) tree analysis was conducted using RAxML v.7.4.2 with 1000 bootstrap replicates and the GTR + I+Gamma model (Stamatakis 2006).

## Results

**Figure 2.**
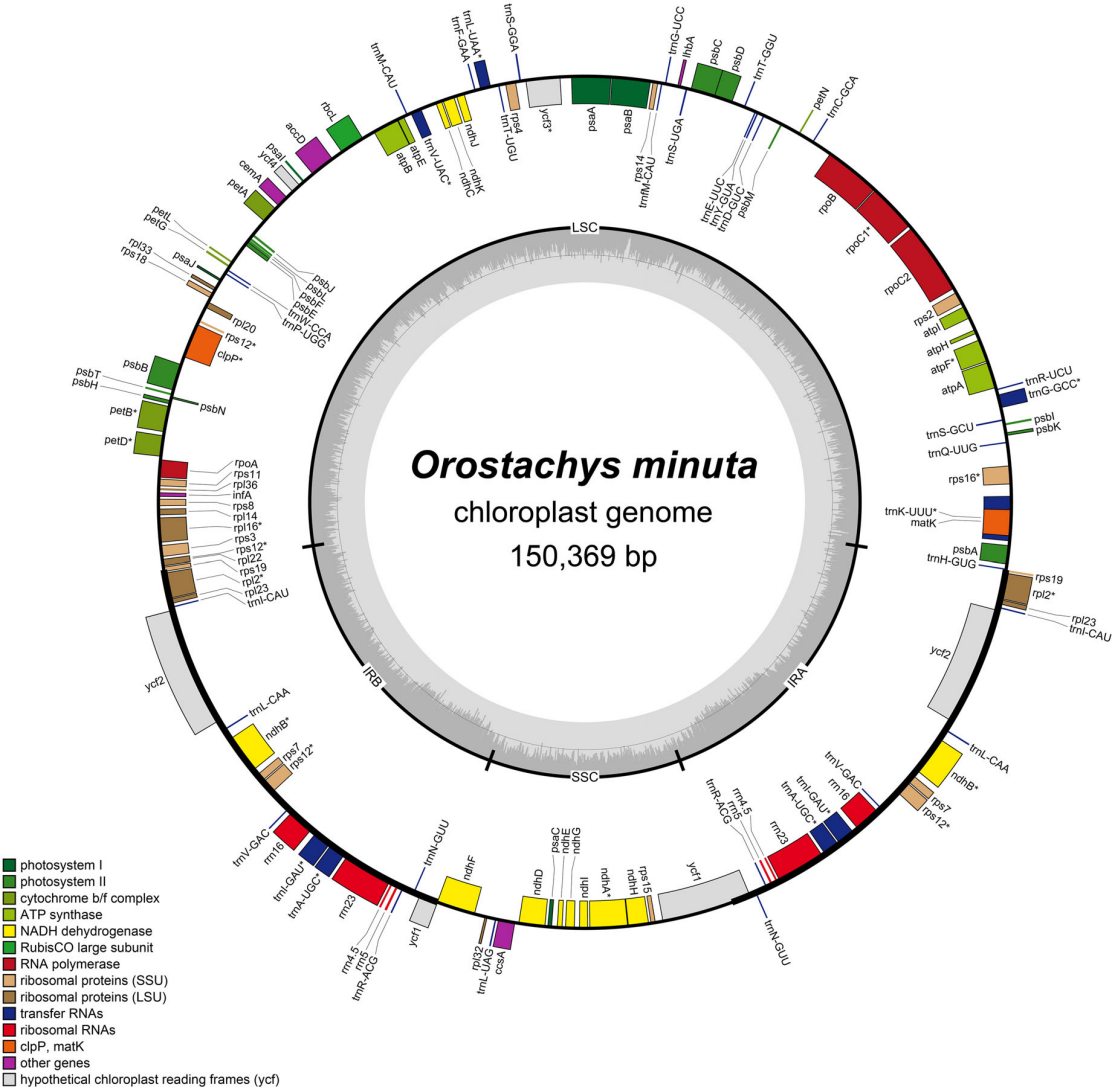
Gene map of the *Orostachys minuta* chloroplast genome. Genes inside the circle are transcribed clockwise, and genes outside are transcribed counterclockwise. The dark grey inner circle corresponds to the GC content, and the light-grey circle corresponds to the AT content.

The complete cp genome of *O. minuta* is a circular DNA molecule of 150,369 bp in length with G + C content of 37.7% composed of a large single-copy (LSC) region of 82,795 bp, a small single-copy (SSC) region of 16,854 bp, and two inverted repeats (IRs) of 25,360 bp (Figure 2). The cp genome contains a total of 113 unique genes (79 protein-coding genes (PCGs), 30 tRNAs, and four ribosomal RNAs (rRNAs)), and 19 genes are duplicated in IR regions (eight PCGs, seven tRNAs, and four rRNAs). Additionally, 18 genes (*atpF*, *clpP*, *ndhA*, *ndhB*, *petB*, *petD*, *rpl2*, *rpl16*, *rpoC1*, *rps12*, *rps16*, *trnA-UGC*, *trnG-GCC*, *trnI-GAU*, *trnK-UUU*, *trnL-UAA*, *trnV-UAC*, and *ycf3*) contain one or two introns.

**Figure 3.**
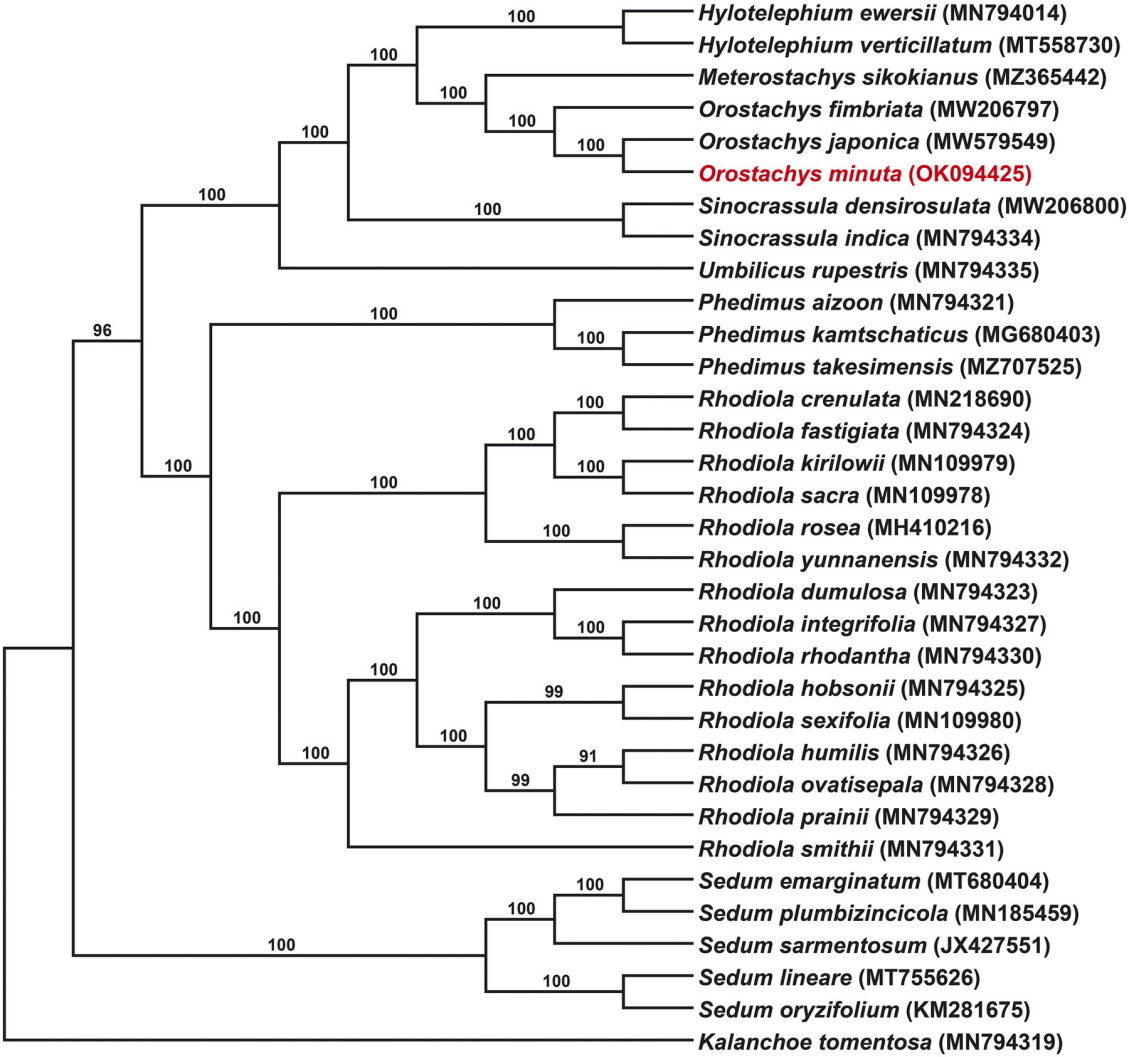
ML tree for *Orostachys minuta* based on complete chloroplast genome sequences. *Kalanchoe tomentosa* (MN794319) was used as outgroup. Numbers at nodes are bootstrap support values in %.

The ML tree formed three clades (Figure 3). The first clade consisted of *Sedum* alone, and the second clade consisted of *Phedimus* and *Rhodiola*, with *Phedimus* forming the most basal part and found to be a sister to all *Rhodiola* species. The last clade comprised *Umbilicus*, *Sinocrassula*, *Orostachys*, *Meterostachys*, and *Hylotelephium*. Genus *Orostachys* was monophyletic in which *O. minuta* was sister to *O. japonica*.

## Discussion

The ML tree was well supported at the genus level and *Orostachys* was monophyletic. The three *Orostachys* species discussed in this study, *O. fimbriata*, *O. japonica*, and *O. minuta*, have spiny apices, and included in subsect. *Appendiculatae* of the two subsections of *Orostachys* (Fu and Ohba 2001; Ohba 2003). This subsection formed the closest phylogenetic relationship to *Meterostachys* in this study, which is the same results as in the previous studies (Gontcharova et al 2006; Messerschmid et al. 2020). This is the first analysis of *O. minuta* in a phylogenetic study. In the result of this study, *O. minuta* showed the closest relationship to *O. japonica* (Figure 3). Morphologically, this species is very similar to *O. japonica* except for its small size, so it is judged that these morphological characteristics were well reflected in our phylogenetic tree.

## Author contributions

All authors contributed to this article, methodology, investigation, software, and writing-original draft, Ha-Rim Lee. Investigation, software, validation, and formal analysis, Bo-Yun Kim, Halam Kang, and Sung-Mo An. Data curation, validation, and writing-review and editing, Kyung-Ah Kim. Conceptualization, validation, writing-original draft, and writing-review and editing, Kyeong-Sik Cheon. All authors agree to be accountable for all aspects of the work.

## Data Availability

The genome sequence data that support the findings of this study are openly available in GenBank of NCBI at https://www.ncbi.nlm.nih.gov/ under the accession no OK094425. The associated BioProject, SRA, and Bio-Sample numbers are PRJNA821378, SRR18558250, and SAMN27068264, respectively.
